# Antioxidant Activity of Coumarins and Their Metal Complexes

**DOI:** 10.3390/ph16050651

**Published:** 2023-04-26

**Authors:** Lozan Todorov, Luciano Saso, Irena Kostova

**Affiliations:** 1 Department of Chemistry, Faculty of Pharmacy, Medical University-Sofia, 1000 Sofia, Bulgaria; irenakostova@yahoo.com; 2 Department of Physiology and Pharmacology “Vittorio Erspamer”, Faculty of Pharmacy and Medicine, Sapienza University, 00185 Rome, Italy

**Keywords:** antioxidant, coumarins, radicals’ scavenging, antioxidant assays, coordination compounds, molecular hybrids

## Abstract

Ubiquitously present in plant life, coumarins, as a class of phenolic compounds, have multiple applications—in everyday life, in organic synthesis, in medicine and many others. Coumarins are well known for their broad spectrum of physiological effects. The specific structure of the coumarin scaffold involves a conjugated system with excellent charge and electron transport properties. The antioxidant activity of natural coumarins has been a subject of intense study for at least two decades. Significant research into the antioxidant behavior of natural/semi-synthetic coumarins and their complexes has been carried out and published in scientific literature. The authors of this review have noted that, during the past five years, research efforts seem to have been focused on the synthesis and examination of synthetic coumarin derivatives with the aim to produce potential drugs with enhanced, modified or entirely novel effects. As many pathologies are associated with oxidative stress, coumarin-based compounds could be excellent candidates for novel medicinal molecules. The present review aims to inform the reader on some prominent results from investigations into the antioxidant properties of novel coumarin compounds over the past five years.

## 1. Introduction

Free radicals are a type of reactive species (RS), bearing an unpaired electron. They are indelibly connected with the processes of life [1]—breathing, metabolism, cell signaling [2] and many others (some prominent examples are presented in Table 1). For that reason, they are constantly being produced in living bodies. Such species include superoxide ion-radicals, hydroxyl radicals, hydrogen peroxide and hydroperoxyl radicals to name a few. Their impact on biological systems can be both helpful and deleterious [3,4]. Maintaining a balance between radical generation and radical elimination is a necessary requirement for good health. Disruption of that balance, caused by insufficient radical elimination, results in oxidative stress, associated with numerous pathologies—cancer [5], diabetes [6], neurodegenerative [7] and coronary artery diseases [8] and many others. That is due to the fact that free radicals are highly reactive, easily interacting with biomolecules and disrupting vital processes. Within the living body exist complex systems of free radical regulation that take into account the particular needs of the immune system and redox signaling [9]. Antioxidant defenses include both endogenous and exogenous substances. Some of these compounds are able to directly scavenge free radicals, a process that involves irreversible chemical transformation of the scavenging molecule. Others can act as chelators, preventing transition metal ions from participating in electron transfer processes, e.g., Fenton-like catalytic radical generation and subsequent peroxidation processes. A class of exogenous compounds that are associated with both scavenging and chelating antiradical action, as well as ROS-producing enzyme inhibition, are coumarins [10].

Coumarin (2H-Chromen-2-one) is the most “basic” member of the eponymous group of phenolic compounds. Historically, coumarins have been applied for the treatment of a variety of diseases due to their anticoagulant, anti-inflammatory, antiviral, antimicrobial, anticancer, antioxidant, etc. activities [11]. That broad spectrum of physiological effects is due to the possibility of a great variety of substitution patterns in the coumarin base structure. Generally speaking, hydrogen-donating substitutions (phenolic, amino, thiol-groups, etc.) of the benzene component of the coumarin “skeleton” tend to improve the antioxidant effect. Ortho-diacetoxy derivatives have also been reported to be effective scavengers [12]. Catecholic motifs are associated with an improvement of antioxidant action [13]. That is due, on one hand, to increased direct scavenging of radicals due to electron donation from the ortho-hydroxyl group and, on the other, bidentate metal chelation which interrupts metal ion-induced reactive species generation [14]. A number of excellent reviews have been published over the years, elucidating the structure–activity effects of coumarin substances [12,13,15,16,17].

The present review focuses on the latest data, regarding the antioxidant properties of coumarin-based molecules and coordination complexes over the past five years. Numerous experiments into modification of the coumarin core have been reported. A subject of particular interest is the design of novel synthetic coumarins, aiming to combine the intrinsic properties of the constituting building blocks in order to produce more potent, combined or even entirely new physiological effects [18]. Another rapidly developing field of research involves coumarin-bearing coordination complexes [19,20]. Metal chelation may not only serve to “isolate” metal ions, thus preventing free radical generation, but could also modify the radical-scavenging properties of the ligands themselves, thus yielding novel complexes with strong antiradical, protective action. As the present review is entirely focused on presenting novel results on coumarins with antioxidant activity, herewith one would find numerous compounds with very disparate physiological activities—antifungal, antiviral, antidepressant, anticoagulant, anticancer, etc. The authors have classified this diverse multitude of chemicals into two, admittedly somewhat arbitrary, groups—molecular coumarin derivatives and coumarin-incorporating complexes.

## 2. Antioxidant Properties of Molecular Coumarins

In terms of molecular structure, the authors have grouped the compounds discussed herein into several subsections: coumarins substituted with small functionalities (hydroxy, methoxy, nitro, methyl groups, etc.); coumarins substituted at positions 3 and/or 4; coumarins, substituted at positions 7 and/or 8; coumarin-imbued polymers; and novel coumarins of natural origins.

### 2.1. Coumarins Substituted with Small Functionalities

Prahadeesh and colleagues synthesized simple coumarins: coumarin, 4-hydroxycoumarin, 7-hydroxycoumarin and 7-hydroxy-4-methylcoumarin [21]. These uncomplicated compounds were tested for peroxide scavenging (direct measurement of H_2_O_2_ in the presence of the compounds, 10 min of incubation) and ferric-reducing antioxidant power (FRAP). The results show that adding a hydroxyl group in position 7 improves peroxide scavenging more than threefold under the experimental conditions (IC_50_ drops from 24,902 mg/L for coumarin to 7029 mg/L for the 7-hydroxy derivative). Hydroxyl group in position 4 increases peroxide scavenging (IC_50_ = 9150 mg/L), though not as much as in position 7. Adding a methyl group at position 4 in addition to the hydroxyl group at position 7 decreases peroxide scavenging (IC_50_ = 11,014 mg/L). The standard substance used for comparison is ascorbic acid, with IC_50_ = 286 mg/L. In terms of ferric-reducing capacity, these substances’ effectiveness decreases in the same order (7-hydroxy > 4-hydroxy > 7-hydroxy-4-methyl > unsubstituted coumarin). This simple experiment, involving uncomplicated structures and easy to understand model systems, serves as an excellent introduction to basic structure–activity relationships when it comes to antioxidant properties of coumarins.

**Figure 1:**
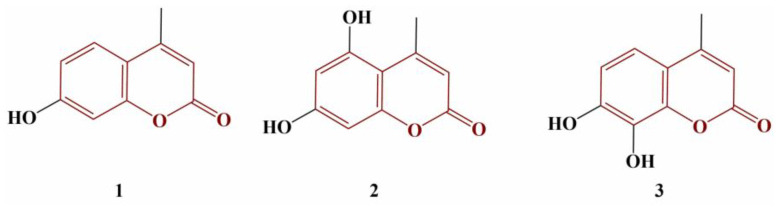
The 4-methylcoumarins, presented in [22].

Couttolenc and colleagues performed a similar structure–activity relationship experiment on three hydroxy-substituted 4-methylcoumarin molecules (Figure 1) [22].

The 2,2-Diphenyl-1-picrylhydrazyl (DPPH), 2,2′-azino-bis(3-ethylbenzothiazoline-6-sulfonic acid) (ABTS) and galvinoxyl radical-scavenging were estimated. Compound 2 was the strongest scavenger of ABTS (EC_50_ = 30.83 μM), followed by Compound 3 (EC_50_ = 39.98 μM). Compound 1 had significantly lower ABTS-scavenging activity (EC_50_ = 1150 μM). EC_50_ of the standard substance Trolox was 83.50 μM. In terms of the DPPH and galvinoxyl assays, Compound 3 again performed better (EC_50_ = 150.99 μM and 13.19 μM, respectively) than Trolox (EC_50_ = 243.39.99 μM and 20.86 μM, respectively). Compounds 1 and 2 did not perform well in these stable radical model systems, with EC_50_s above 2400 μM. The authors attribute the observed differences on the number and positioning of the hydroxyl groups. Compound 3 allows for an intramolecular hydrogen bond that stabilizes the semiquinone radical and thus improves scavenging activity [23].

**Figure 2:**
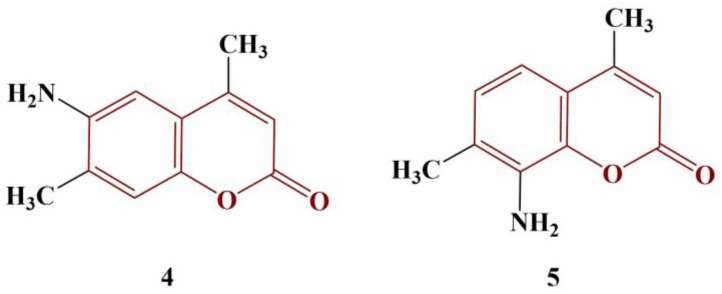
The two most active scavengers, described in [24].

A number of novel methyl-, nitro- and/or amino-substituted coumarin derivatives have been synthesized and tested for radical scavenging activity against DPPH (methanol, 1 h incubation, 37 °C) [24]. Two of the compounds, presented in Figure 2, behaved as strong scavengers.

Compound 4 (IC_50_ = 10 μg/mL) was more active than ascorbic acid (IC_50_ = 33.48 μg/mL). The potency of Compound 5 was lesser than that of the standard (IC_50_ = 42.90 μg/mL). Two electron-donating amino groups at positions 6 and 8 dramatically decrease the antioxidant effect (IC_50_ = 110.20 μg/mL). Exchanging the amino groups for electron-withdrawing nitro groups further decreases DPPH scavenging activity.

### 2.2. Coumarins, Substituted at Positions 3 and/or 4

**Figure 3:**
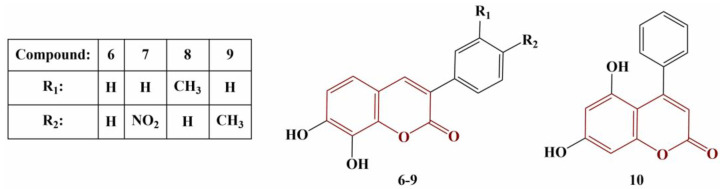
The hydroxycoumarins described in [25].

Ozalp and colleagues synthesized a series of hydroxycoumarins (Figure 3) and tested them for DPPH scavenging, FRAP, cupric-reducing antioxidant capacity (CUPRAC) and metal chelating [25].

In terms of DPPH scavenging, all 7,8-dihydroxy members manifested significant activity (EC_50_ < 94.85 μM), equal to or higher than that of the standard compound Trolox (EC_50_ = 93.19 μM). Compound 10 had very low activity with EC_50_ = 6604.92 μM. Most active were Compounds 6 and 7 (EC_50_ = 74.70 and 64.27 μM, respectively). Compound 7 also exhibited the highest ferric-reducing and copper-reducing potentials (EC_50_ = 2.28 μM and 2.44 μM, respectively, versus EC_50_ = 1.0 μM for Trolox in both assays). Inversely, the 5,7-dihydroxo-substituted member of the series had the best ferrous-ion-chelating activity (EC_50_ = 2.702 mM), compared to Compounds 6–9 (EC_50_ = 0.728 mM up to 0.782 mM) and Trolox (EC_50_ = 1.191 mM).

**Figure 4:**
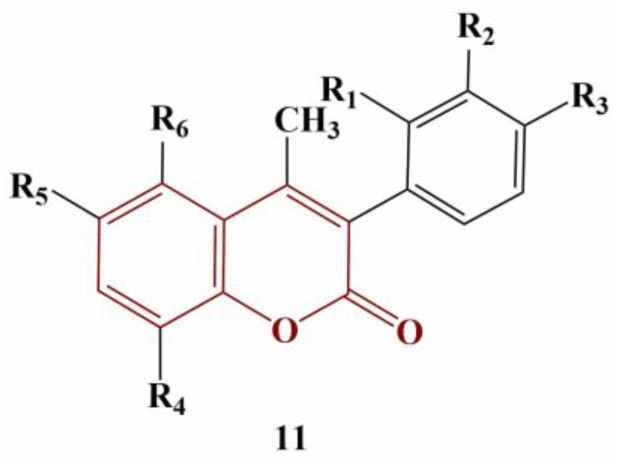
Common structure of the 3-phenylcoumarins described in [26].

Katopodi and colleagues synthesized a number of multisubstituted 3-phenylcoumarins [26]. Their common structure is presented in Figure 4.

All coumarin analogues were investigated with a panel of tests—ABTS, suppression of Fenton-generated hydroxyl radicals (DMSO competition), inhibition of 2,2′-azobis-2-methyl-propanimidamide (AAPH)-induced linoleic acid oxidation and 2′,7′-dichlorodihydrofluorescein diacetate (DCFDA) assay. All assays were performed with 100 μM concentrations of the tested compounds, with Trolox and ascorbic acid being used for comparison. Results were presented as percentage of inhibition at 100 μM. Most compounds did not reduce ABTS or did so to a very limited extent (0 to 27.8%). Adding a hydroxyl group at position 5 of the coumarin structure (R6) significantly improves activity in this model system (49.2 to 73.3%). If R3 is a hydroxyl group, ABTS scavenging is negated. An acetyloxy group at R3 or R6 improves scavenging of hydroxyl radicals and suppresses AAPH-induced oxidation. Two acetyloxy groups at R2 and R3 decrease that effect. Adding chlorine or fluorine at R1 or R2 increases scavenging of hydroxyl radicals, but the same substitution at R3 causes the opposite effect. A hydroxyl group or a fluorine atom at R3 decreases peroxide-induced intracellular ROS (as per the DCFDA assay) by 100%.

**Figure 5:**
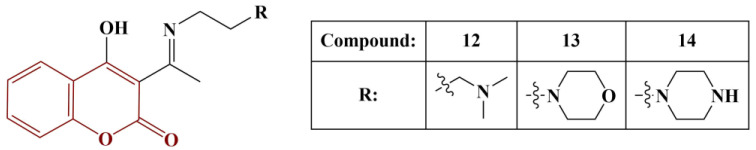
The Schiff-base-infused coumarins, described in [27].

A series of novel 4-hydroxycoumarin derivatives (Figure 5) were synthesized and tested for antioxidant activity as part of an effort to synthesize compounds with anti-Alzheimer’s activity [27]. The authors aimed to improve metal chelation by adding Schiff base functionality in addition to the phenolic hydroxyl group. A tertiary amine was added to improve anticholinergic activity and lipid–water partition coefficient.

Radical scavenging was assessed by way of Fenton-generated hydroxyl (OH) radicals and cyclic voltammetry (for assessing superoxide scavenging). All compounds manifested significant hydroxyl scavenging with IC_50_ between 2.61 and 2.94 μM. Compound 13 was the strongest scavenger and was consequently complexed with Cu(II). The novel complex was even more active with IC_50_ = 0.20 μM. The standard compound ascorbic acid had IC_50_ > 300 μM. Cyclic voltammetry showed concentration-dependent superoxide scavenging. In this case, Compound 12 seemed to completely eliminate superoxide in the model system at 5 mM.

**Figure 6:**
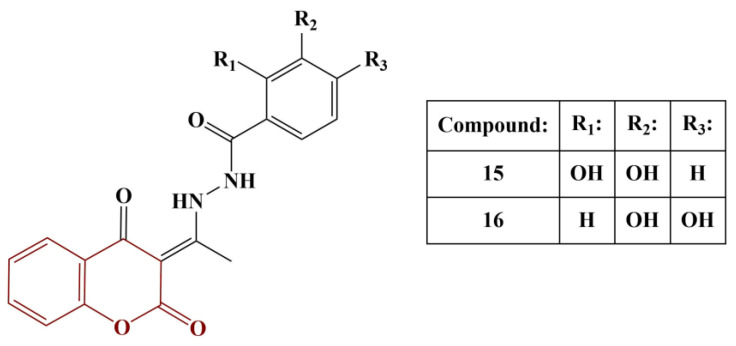
The coumarin–benzohydrazides described in [28].

Antonijevic and colleagues synthesized a series of coumarin–hydroxybenzohydrazide hybrids and investigated their potential antioxidant properties in vitro [28]. Additionally, theoretical investigations into the mechanisms of their activity were performed with the aid of DFT. Synthesis involved condensation of 3-acetyl-4-hydroxycoumarine with appropriate hydrazides (acetic acid as catalyst) in ethanol (heating) or n-propanol (reflux). Antioxidant activity was established with the aid of the DPPH assay. The two most active members of the series are presented in Figure 6.

Compounds 15 and 16 had IC_50_ of 2.9 ± 0.1 μM and 12.9 ± 0.4 μM, respectively. IC_50_ of the positive controls nordihydrohuaiaretic acid (NDGA) and quercetin were calculated as 1.7 ± 0.1 μM and 1.9 ± 0.1 μM, respectively. The significant antioxidant activity of these two substances was attributed by the authors to the presence of two neighboring phenolic groups. Hydrogen atom transfer (HAT) and sequential proton loss electron transfer (SPLET) mechanisms were found to be most probable in non-polar and polar solvents, respectively.

**Figure 7:**
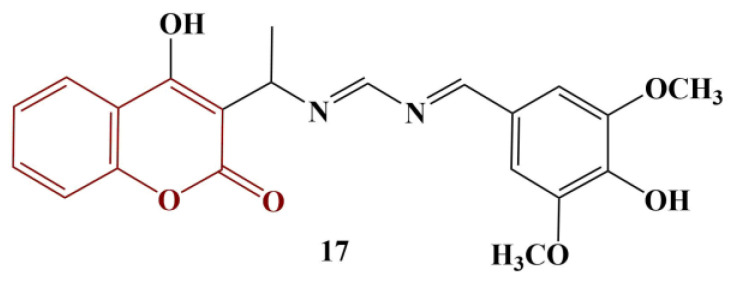
The most active asymmetric azine, described in [29].

A group of asymmetric azines containing a 4-hydroxycoumarin moiety were synthesized and tested for scavenging activity toward DPPH [29]. The authors claim that most of the tested compounds did not behave as antioxidants to a significant extent, despite the presence of a hydroxyl group at position 4 in the coumarin functionality. The only exception, presented in Figure 7, behaved as a strong, fast scavenger of DPPH (EC_50_ = 0.53, presented as moles of antioxidant per 1 mol DPPH against EC_50_ = 0.25 for ascorbic acid).

The authors proposed that the overall passivity of the hydroxyl group in position 4 was due to intramolecular hydrogen bonding. The “exceptional” compound could attribute its antioxidant activity to the hydroxyl group, present in the phenyl moiety, additionally “supported” by the neighboring electron-donating methoxy groups.

**Figure 8:**
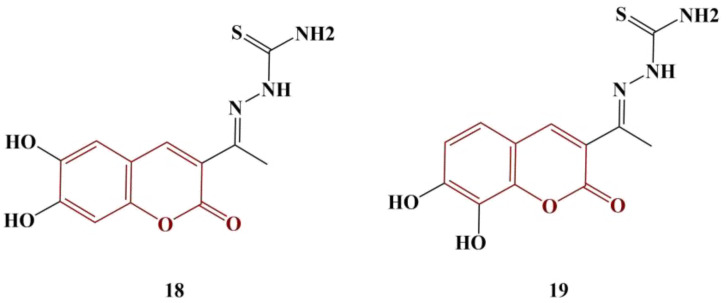
The most active coumarin–thiosemicarbazones, described in [30].

Coumarin–thiosemicarbazones were synthesized as potential antityrosinase agents and tested for antioxidant action [30]. Two of the members (Figure 8), bearing a catecholic motif in the coumarin fragment, manifested significant antioxidant activity.

DPPH and ABTS testing results showed that Compound 18 (IC_50_ = 7.1 μM and 9.0 μM, respectively) and Compound 19 (IC_50_ = 17.9 μM and 8.8 μM, respectively) performed better than both standard compounds—ascorbic acid (DPPH assay IC_50_ = 18.6 μM) and Trolox (ABTS assay, IC_50_ = 13.0 μM). The other members of the series bore a single hydroxyl substituent in their coumarin fragment and had more moderate scavenging effect.

**Figure 9:**
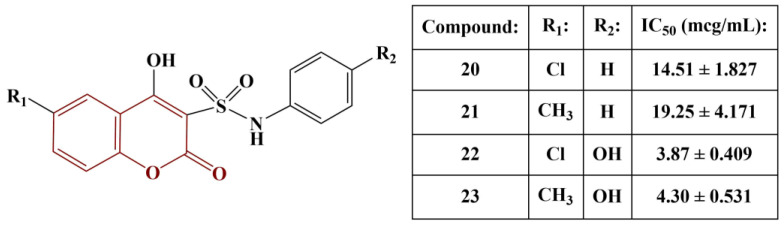
The active coumarin-3-sulfonamides described in [31].

A series of pyranocoumarins, coumarin-3-sulfonamides and coumarin-sulfonamide-chalcones have been synthesized and tested for antioxidant activity using the DPPH (0.05 mM) assay [31]. Pyranocoumarins exhibited very low or zero activity. The same was observed in the chalcone-substituted compounds. The most potent scavengers of DPPH are presented in Figure 9. Ascorbic acid was used for comparison (IC_50_ = 2.83 μg/mL)

The chlorine atom at position 6 of the coumarin structure improves scavenging activity compared to a methyl group in the same place. Adding a phenolic hydroxyl group to the benzene ring significantly improves activity. Notably, compounds 20–23 were also found to exhibit significant COX1/2-inhibitory activity as well, similar to Indomethacin and Celecoxib. When considering the rest of the compounds, synthesized by the authors, in terms of structure–activity relationships, sulfathiazole- and sulfanilamide-containing compounds showed higher activity than sulfadiazine-substituted substances.

**Figure 10:**
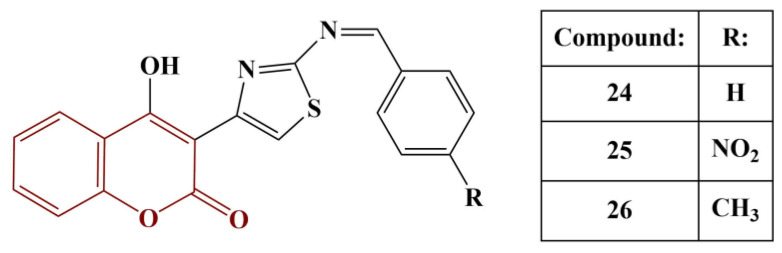
The most active thiazolyl-coumarins described in [32].

New thiazolyl-coumarin derivatives were synthesized and tested for antioxidant activity (DPPH test, 0.1 mM) [32]. The most active compounds from the series are presented in Figure 10.

Compounds 24–26 scavenged DPPH to the same extent as gallic acid and butylated hydroxytoluene (BHT) across all tested concentrations. Adding electron-donating (hydroxyl and methoxy) groups to the side benzene ring decreases antioxidant activity in this model system. The authors also propose that the hydrazinothiazole functionality plays an important part in the antioxidant action.

**Figure 11:**
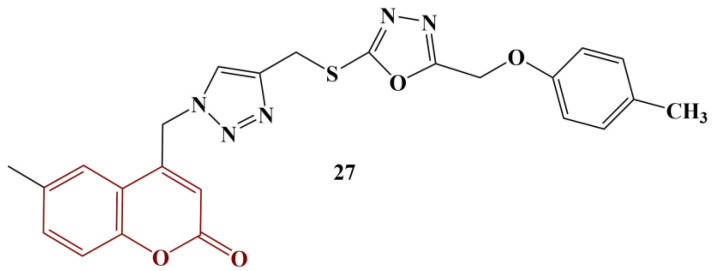
The most active coumarin hybrid, described in [33].

Kumar and colleagues synthesized a series of hybrids, merging 1,2,3-triazoles, 1,3,4-oxadiazole and coumarins [33]. They were tested for scavenging of DPPH (ethanol, 30 min incubation, room temperature). One of the novel compounds (Figure 11) manifested moderate scavenging activity (28.2% inhibition) at a concentration of 40 μg/mL.

Exchanging the 4-methyl group of the phenyl ring for a chlorine atom decreases scavenging to 18.2%. Adding to it a methyl group at the meta-position almost negates any activity.

Naik and colleagues synthesized a number of 3-substituted coumarin–amino acid hybrids [34]. Scavenging of DPPH (0.004% in ethanol, 30 min of incubation in dark) and nitric oxide (sodium nitroprusside with Griess reagent, 150 min incubation at room temperature) were assessed. These hybrids were generated by coupling of coumarin-3-carboxylic acid with esters of several amino acids. In terms of the DPPH assay, the best results were obtained with the coumarin–tyrosine (IC_50_ = 31.45 μg/mL) and coumarin–serine (IC_50_ = 28.23 μg/mL) hybrids compared to the standard substance (ascorbic acid, IC_50_ = 20.53 μg/mL). All compounds scavenged nitric oxide, the least pronounced effect being observed with the glycine and phenylalanine conjugates (IC_50_ = 96.94 μg/mL and IC_50_ = 66.20 μg/mL, respectively). The presence of aliphatic or aromatic hydroxyl groups increased the observed scavenging effect. For example, the IC_50_ of the tyrosine hybrid was 26.90 μg/mL. Ascorbic acid had IC_50_ = 18.40 μg/mL.

A coumarin–benzothiazole hybrid was synthesized and tested for antioxidant action with DPPH (0.2 mM, DPPH, 20 min cultivation, 28 °C) [35]. Results were compared to ascorbic acid. The novel hybrid scavenged DPPH with an IC_50_ = 591.58 μg/mL. The result for the standard compound was IC_50_ = 391.25 μg/mL.

**Figure 12:**
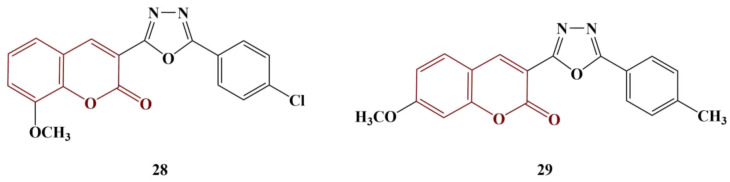
The most active coumarin–oxadiazole hybrids, described in [36].

Coumarin-tethered 1,3,4-oxadiazole analogues [36] were synthesized and tested as potential scavengers of DPPH and hydroxyl radicals. Out of ten molecules in total, two manifested significant antioxidant activity (Figure 12, Compounds 28 and 29).

The DPPH assay involved 30 min incubation at 37 °C of a number of concentrations of each substance in 0.4 mM DPPH. Compounds 28 and 29 manifested DPPH-scavenging activity with IC_50_s of 19.47 μM and 17.19 μM, respectively. For ascorbic acid, IC_50_ = 23.80 μM. Scavenging of hydroxyl radicals was estimated using Fenton-reaction degradation of 2-deoxyribose (60 min incubation at 37 °C), followed by derivatization with thiobarbituric acid (TBA) (15 min, boiling water). Butylhydroquinone (BHA) was used as a standard. Compounds 28 and 29 manifested OH-scavenging activity with IC_50_s of 32.62 μM and 28.51 μM, respectively. These results demonstrated similar activity to BHA (IC_50_ = 36.05 μM). Overall, improvement of the activity in both model systems was associated with adding a methoxy group in positions 7 and 8, as well as adding a chlorine atom at position 6, of the coumarin structure.

**Figure 13:**
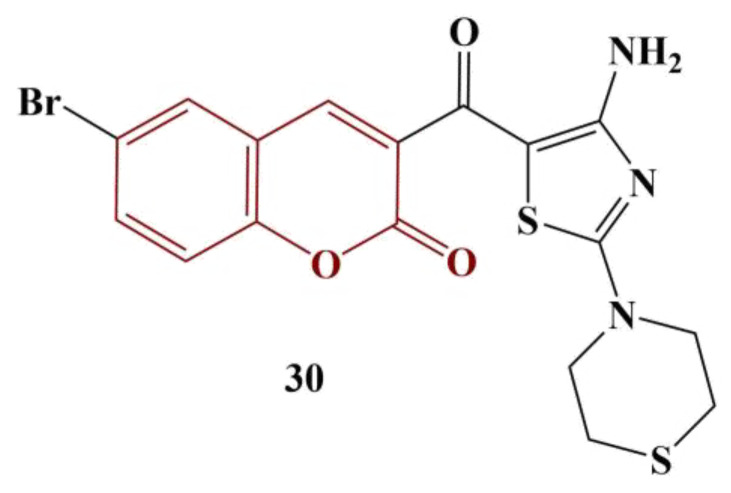
The most active coumarin–diaminothiazole, described in [37].

A series of novel coumarins bearing a 2,4-diaminothiazole-5-carbonyl moiety were synthesized and tested for DPPH-scavenging ability [37]. Almost all compounds manifested good scavenging ability, with IC_50_ < 50 μg/mL. The most active substance (Figure 13) had IC_50_ = 23.9 ug/mL—about three times higher than that of the standard substance ascorbic acid.

Compounds with morpholine, piperidine and pyrrolidine moieties attached to the thiazole ring improved radical elimination. The introduction of Br at position 6 of the coumarin structure tended to diminish DPPH scavenging.

**Figure 14:**
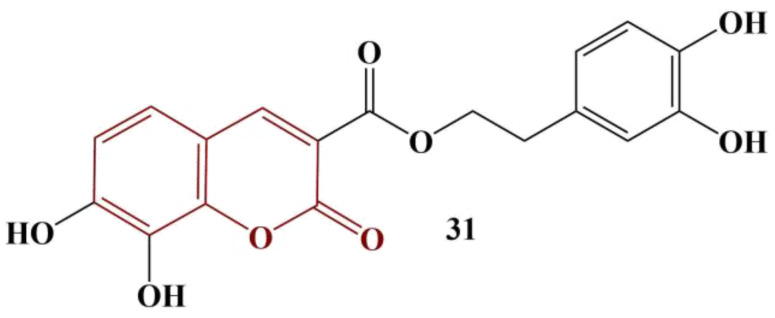
The most active coumarin–hydroxytyrosol hybrid, described in [38].

Li and colleagues synthesized conjugates of coumarin and hydroxytyrosol, a proven natural antioxidant. DPPH and ABTS assays were utilized to assess antioxidant activity, BHT being the control substance [38]. The most potent compound is presented in Figure 14.

Coumarin itself has extremely low scavenging activity (IC_50_ > 10 000 μM) in both model systems. Hydroxytyrosol manifested moderate activity (DPPH IC_50_ = 143.81 μM, ABTS IC_50_ = 170.47 μM). BHT had DPPH IC_50_ = 521.99 μM and ABTS IC_50_ = 127.07 μM. Conjugating a coumarin to hydroxytyrosol caused improved scavenging activity in both model systems. The most active substance (Figure 14) had DPPH IC_50_ = 26.58 μM and ABTS IC_50_ = 30.31 μM. In terms of structure–activity relationships, the authors noted that methyl, methoxy and chlorine substitutes at positions 6, 7 and/or 8 of the coumarin structure slightly improve DPPH scavenging, but diminish ABTS scavenging. Substituting hydroxyl groups at these locations improves scavenging activity in both model systems with the 7,8-dihydroxy-substituted member being the most active antioxidant.

### 2.3. Coumarins, Substituted at Positions 7 and/or 8

**Figure 15:**
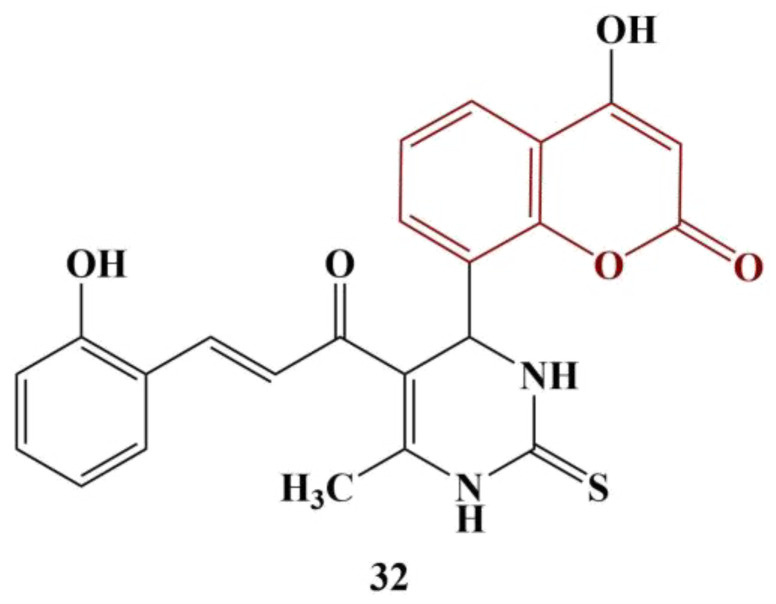
The most active coumarin–chalcone hybrid, described in [39].

Konidala and colleagues synthesized 4-hydroxycoumarin–chalcone molecular hybrids [39] and tested them for their antimicrobial and antioxidant properties. Antioxidant activity was characterized with the aid of the DPPH assay. All compounds were tested at a single concentration (100 μg/mL). Results were presented as scavenging percentages compared to negative control (no substance present). Ascorbic acid (81.21% scavenging) and BHT (70.05% scavenging) were used for comparison. All compounds behaved as moderate scavengers of DPPH. The most active one is presented in Figure 15 (77.92% scavenging).

Removing the hydroxyl group from the benzene ring slightly diminishes radical scavenging (75.22%). Moving it from the ortho- to meta-position decreases activity by more than half; a para-positioned hydroxyl group causes even further diminishing of activity. A single methoxy functionality at the para-position decreases activity (31.28% scavenging). Three methoxy groups at meta- and para-positions improve activity (71.32% scavenging).

De Souza and colleagues synthesized a series of 3-(4-(dimethylamino)phenyl)-7-aminoalcoxycoumarins [40]. They tested them for ferric-reducing potential. What they discovered was that the number of carbon atoms separating nitrogen and oxygen in the aminoalcoxy substituent impacts ferric-reducing ability. When that chain includes one carbon atom, FRAP IC_50_ was 7.49 mmol Q(quercetin)/mol of tested compound. Increasing the chain’s length to two and three carbon atoms decreases FRAP values threefold.

A series of coumarin–rasagiline hybrids underwent a spectrum of tests to assess their neuroprotective, MAO-B-inhibitory and radical-scavenging properties [41]. The novel molecules were functionalized with propargyl groups in positions 3, 4 or 7 of the coumarin structure. DPPH assay (50 μM in ethanol, 30 min incubation at room temperature) yielded moderate antioxidant activities of all compounds (100 μM concentrations). Scavenging activities varied between 10–20%, with few statistically significant differences and no clear structure–activity relationship to be observed.

Joy and colleagues investigated a number of 4-methyl-7-alkynyl coumarin derivatives [42] with the DPPH assay (0.004%, 10 min cultivation, room temperature, dark conditions). BHT was used for comparison. Results were presented as percentage inhibition compared to negative control (no substance present). Adding a hydroxymethyl, 4-hydroxyphenyl or 4-aminophenyl moiety to the alkynyl substituent yields significant scavenging activity (63,3%, 74,2% and 70,8% at 100 μg/mL concentration). BHT scavenged about 90% of DPPH at 100 μg/mL. The authors propose that the incorporation of electron-donating groups stabilizes the oxygen-centered radical, thus improving the ability of the respective compound to donate a hydrogen atom to DPPH.

**Figure 16:**
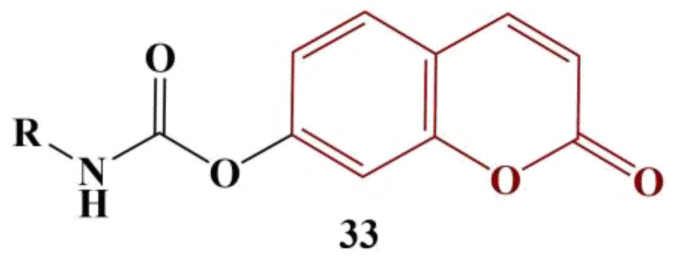
The coumarin–carbamates, described in [43].

Kurt and colleagues synthesized a series of carbamate-substituted coumarins [43], as shown in Figure 16.

When the substitute R is a cyclopentyl, cyclohexyl or cyclohexylmethyl, moderate ABTS scavenging was observed (IC_50_ between 66.80 and 80.03 μM). A cycloheptyl substitute increases IC_50_ to 131.85 μM. Adding a methyl group at position 4 of the coumarin structure tends to decrease the scavenging activity about twofold. A surprising exception is the member in the molecule of which R is a cyclohehyl moiety (IC_50_ = 23.15 μM). Quercetin was used for comparison (IC_50_ = 15.149 μM).

**Figure 17:**
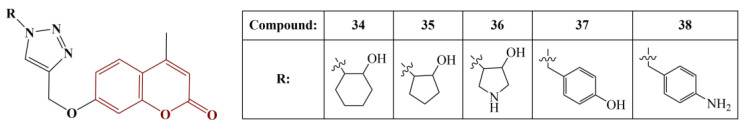
The most active coumarin–triazoles, described in [44].

Joy and colleagues synthesized a series of coumarins, linked to 1,2,3-triazoles, using copper-catalyzed, azide-alkyne cycloaddition [44]. The substances were screened for antimicrobial and antioxidant action. Antioxidant activity was measured with the DPPH assay. BHT was used as a standard. Results were presented as percentage inhibition compared to negative control (no substance present) at 100 μg concentration. The most active antioxidants are displayed in Figure 17.

The hydrogen-donating activity of compounds 34–38 was between 61,8% and 74,2% of that for the standard BHT. The authors propose the improved antioxidant activity of these particular compounds compared to the rest of the series may be due to the electron-donating hydroxyl and amino groups incorporated in the radical R.

Popova and colleagues synthesized novel [1,3]oxazine derivatives of 7-hydroxy-6-isobornyl-4-methylcoumarin [45]. Total antioxidant activity was measured in vitro in terms of the inhibition of Fenton-reaction-induced lipid peroxidation in mouse brain homogenates (1 h incubation at 37 centigrade), followed by TBA reaction. Scavenging of DPPH (methanol solution) was also measured after 30 min of incubation at room temperature in dark conditions. In both cases, Trolox was used as a reference. In terms of DPPH, all novel compounds manifested very low scavenging activity (between 5 and 20 times lower than Trolox) at 10 μM and 100 μM. On the other hand, all of them inhibit lipid peroxidation in mouse brain homogenates to a higher extent than Trolox. The authors propose that the observed effect may be due to the lipophilicity of the substances and the specific properties of the model system—an emulsion that causes molecules to aggregate at the phase boundary. This hypothesis is confirmed by the fact that increasing the hydrophilicity of the compounds causes a decrease in the peroxidation-inhibitory activity.

**Figure 18:**
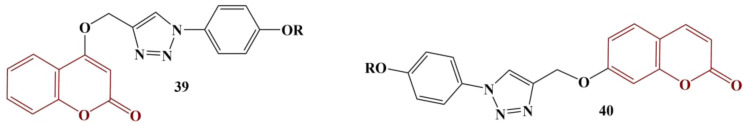
The coumarin–triazole hybrids, described in [46].

A series of coumarin hybrids with 1,2,3-triazoles were synthesized and tested for antioxidant activity (DPPH assay) [46]. Two classes of compounds were generated: 4-substituted and 7-substituted coumarins (Figure 18: 39 and 40).

Both series of compounds were substituted with the same functionalities (a hydrogen atom or a variety of substituted benzyl moieties), allowing for a direct comparison in terms of antioxidant properties of similar substances, differing only in the particular site of coumarin substitution. Generally, the 7-substituted coumarins performed better than their 4-substituted counterparts. Monomethyl-substituted benzyl moieties tended to improve overall antioxidant activity, particularly the meta-substituted ones. Fluorine substitution produced better activity than chloro- or bromo-substitution. Nitro-substitution either did not change or impaired antioxidant activity compared to compounds where the benzyl moiety was unsubstituted. All compounds were less active (IC_50_ between 3.33 and 9.74 μg/mL) compared to the standard ascorbic acid (IC_50_ = 1.23 μg/mL).

**Figure 19:**
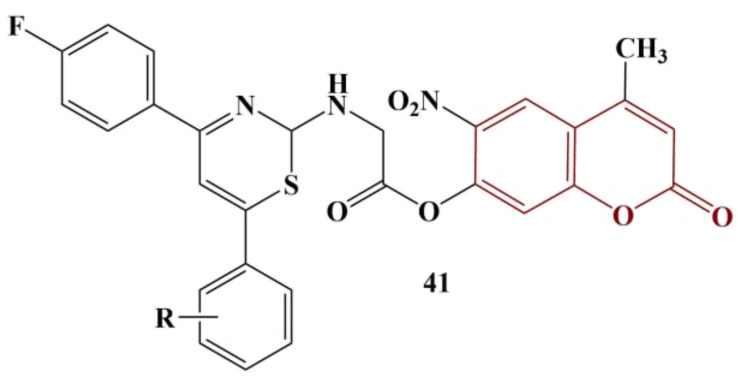
Structure of the thiazine–coumarin hybrids, described in [47].

A series of coumarin-coupled thiazines (Figure 19) were synthesized and underwent antioxidant testing [47]. DPPH (180 μg/mL, methanol, 25 min cultivation) and ABTS were applied. The best results with DPPH were obtained when the moiety R was hydrogen, 2-Cl and 4-Cl (IC_50_ between 35.35 and 40.02 μg/mL; ascorbic acid had IC_50_ = 36.22 μg/mL). Radical-scavenging activity is reduced by 4-OH and 4-F substitution.

On the other hand, 4-F and 4-CH_3_ substitution provided the best result with the ABTS test (IC_50_ = 53.92 and 52.00 μg/mL, respectively; ascorbic acid had IC_50_ = 22.64 μg/mL). Contrary to DPPH, 2-Cl, 4-Cl and hydrogen substitution in the benzene ring decreases ABTS scavenging.

Xue and colleagues performed theoretical investigations of a number of 7-hydroxycoumarin–chalcone hybrids [48]. The chalcone moiety was attached at position 8 of the coumarin structure. The results obtained demonstrated that the 7-OH group in ring B of the coumarin structure is less favorable as a donor of hydrogen atoms compared to differently positioned OH groups in the same ring. Theoretical calculations demonstrated that the position 7 hydroxyl group forms an intramolecular hydrogen bond with the chalcone carbonyl oxygen atom. The presence of such a bond increases the bond-dissociation energy of the 7-OH and makes it less favorable for participation in HAT compared to OH groups at the chalcone benzene ring. In gas phase/benzene medium, the HAT mechanism was calculated to be thermodynamically more favorable.

Gunduz and colleagues synthesized a new coumarin derivative—7-((8-(4-benzylpiperidin-1-yl)octyl)oxy)-4-methyl-2*H*-chromen-2-one [49]. The novel compound was tested for antiproliferative and antioxidant activity. DPPH assay showed moderate activity (concentrations between 0.03125 and 1.0 mg/mL), weaker than that of the standard BHT at the same concentrations. Polar media (ethanol or water) seemed to favor the SPLET mechanism, as solvation was calculated to decrease proton affinities.

### 2.4. Coumarin-Imbued Polymers

**Figure 20:**
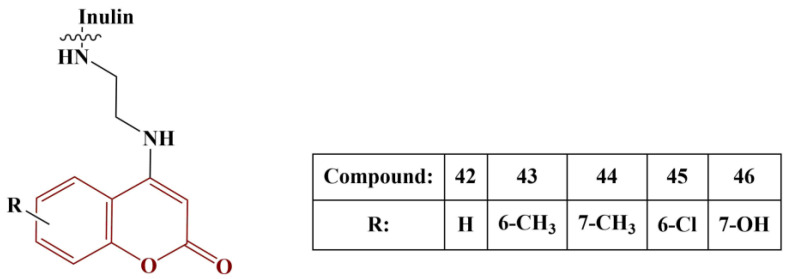
Structure of the coumarin-functionalized inulins described in [50].

Yang and colleagues synthesized a series of coumarin-functionalized inulin derivatives [50]. The coumarin moieties, chemically attached to inulin, are presented in Figure 20.

Superoxide scavenging was measured with the help of 5-methylphenazinium methyl sulfate (PMS)-reduced nicotinamide adenine dinucleotide (NADH) coupling in the presence of nitro blue tetrazolium (NBT) (5 min incubation at room temperature). DPPH assay (180 μM, ethanol) was applied (30 min incubation, room temperature). Ferric-reducing ability was tested with 1% potassium ferricyanide (20 min incubation, 50 °C). Vitamin C was used as reference in all three previous assays. Using vitamin E as a reference, 2-Phenyl-4,4,5,5-tetramethylimidazoline-1-oxyl-3-oxide (PTIO) scavenging was measured (2 h incubation, 37 °C). All tested compounds demonstrated excellent superoxide scavenging. The 7-hydroxy-substituted compound was the most active (IC_50_ = 0.04 mg/mL), followed closely by the 6-chloro-substituted one. DPPH results again showed good scavenging effect (IC_50_ being between 0.08 and 0.11 mg/mL) and, in this case, the 6-chloro-substituted compound was most active. PTIO scavenging of all substances was high as well (IC_50_ being between 0.23 and 0.26 mg/mL) and, in this case, Compounds 43 and 46 were the most active. The ferric-reducing ability of Compounds 43 and 44 was highest, though not as high as that of vitamin C. Overall, the 6-chloro-substituted derivative manifested the most “evenly high” antioxidant effect, followed by the 7-hydroxy derivative.

**Figure 21:**
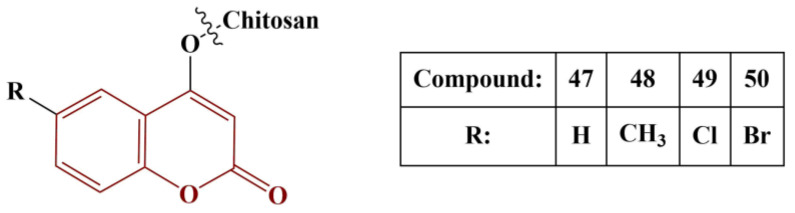
The coumarin–chitosan hybrids, described in [51].

Another interesting experiment involving a coumarin-functionalized polymer was performed by Li and colleagues [51]. Chitosan was imbued with several different coumarins, as presented in Figure 21.

The novel polymers underwent several radical-scavenging assays: DPPH (180 μM in ethanol, 30 min cultivation, room temperature); superoxide (PMS/NADH/NBT, 5 min incubation, room temperature); hydroxyl radical (Fenton reaction in presence of safranine T, 30 min cultivation, 37 °C) and metal chelation (FeCl_2_/ferrozine, 10 min cultivation at room temperature). Within the entire range of assays, the coumarin-modified polymers performed better than unmodified chitosan. Compound 49 was the best scavenger of hydroxyl radicals (IC_50_ = 0.09 mg/mL), followed closely by Compound 50 (IC_50_ = 0.10 mg/mL), meaning that halogenation at position 6 of the coumarin functionality is beneficial. All compounds showed good DPPH scavenging (IC_50_ between 0,11 and 0.14 mg/mL). Promising results were obtained from the superoxide-scavenging assay (IC_50_ between 0.36 and 0.48 mg/mL, the best results being obtained by the halogenated members). The novel polymers showed dramatically improved chelating ability, compared to chitosan (IC_50_ between 0.02 and 0.04 mg/mL). Generally, the polymers, bearing halogenated coumarin, performed best in all tests.

### 2.5. Novel Coumarins of Natural Origin

A natural 7-hydroxycoumarin has been isolated from the fungus *Aspergillus versicolor* [52] and subjected to DPPH (0.16 mM in ethanol) and ABTS assays (6 min incubation, room temperature). The compound behaved as a moderate scavenger (ABTS IC_50_ = 128.8 μM and DPPH IC_50_ = 237.3 μM).

**Figure 22:**
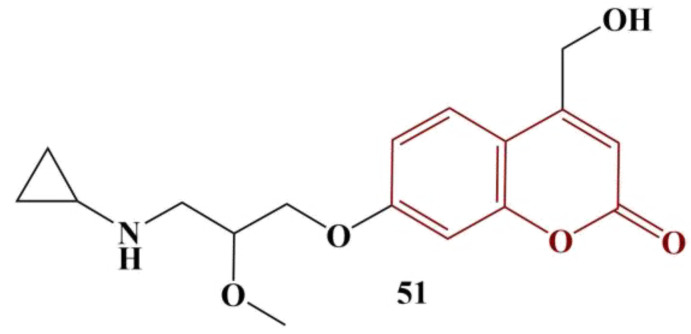
The tested substance, described in [53].

A novel series of coumarins were synthesized by way of biotransformation by *Candida albicans* [53]. Out of six novel compounds, one (Figure 22) was discovered to bear antimicrobial activity after biotransformation and was further studied for antioxidant activity. DPPH assay (2.5 mg/100 mL in ethanol) was performed. Results were measured after 30 min incubation in dark conditions.

The tested compound manifested good antioxidant activity—at 500 μg/mL, DPPH scavenging was 43.5% against 51% for ascorbic acid.

Another naturally occurring coumarin (7-Hydroxy 3-methoxy coumarin 5-O-β-glucopyranoside) was isolated from the Cuphea ignea shrub [54]. The compound was tested with DPPH assay (ethanol, 30 min incubation, 37 °C, dark). The authors report ED_50_ = 6.31 μg/mL. Oxygen radical absorbance capacity (ORAC) assay was additionally performed, yielding an ED_50_ = 5.78 μg/mL, exceeding the result for the positive control Trolox (ED_50_ 27.0 μg/mL).

**Figure 23:**
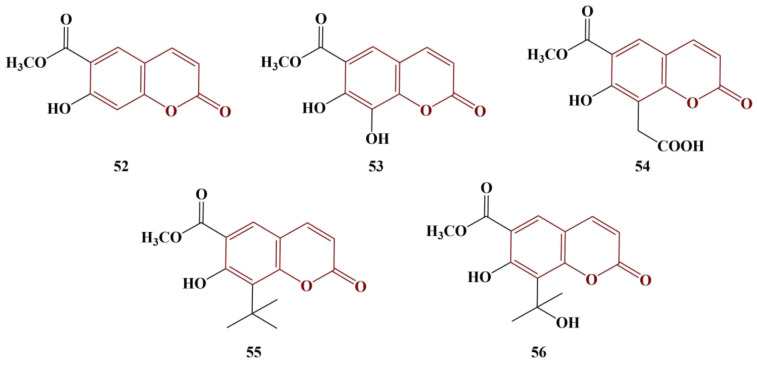
The coumarins, described in [55].

A series of natural coumarins were extracted from Granny Smith apple seeds [55]. Their structures are presented in Figure 23. DPPH (methanol, 30 min incubation in dark at room temperature) and Fenton-generated hydroxyl radical (FeCl_3_, β-phenanthroline and H_2_O_2_, 5 min’ incubation, room temperature) scavenging was measured. As expected, the strongest scavenger in both model systems is the 7,8-dihydroxy-substituted Compound 53 (DPPH IC_50_ = 48.20 μM, hydroxyl radical IC_50_ = 52.84 μM). Ascorbic acid was used for comparison: DPPH IC_50_ = 46.29 μM, hydroxyl radical IC_50_ = 50.33 μM.

Compound 52, which lacks any substituent in position 8, performs the worst, but still seems to be a moderately effective antioxidant (IC_50_ = 103.09 μM and 97.03 μM for DPPH and the hydroxyl radical assays, respectively).

Auraptene, a monoterpene-coumarin ether derived from *Ferula szowitsiana*, was tested for nitric-oxide-scavenging activity [56]. The observed IC_50_ = 670.9 μg/mL demonstrates a moderate scavenging effect. Additional TBA assay reveals 14% suppression of hydroxyl radicals. The authors also noted it upregulates SOD, CAT and GPx in human foreskin fibroblasts.

**Figure 24:**
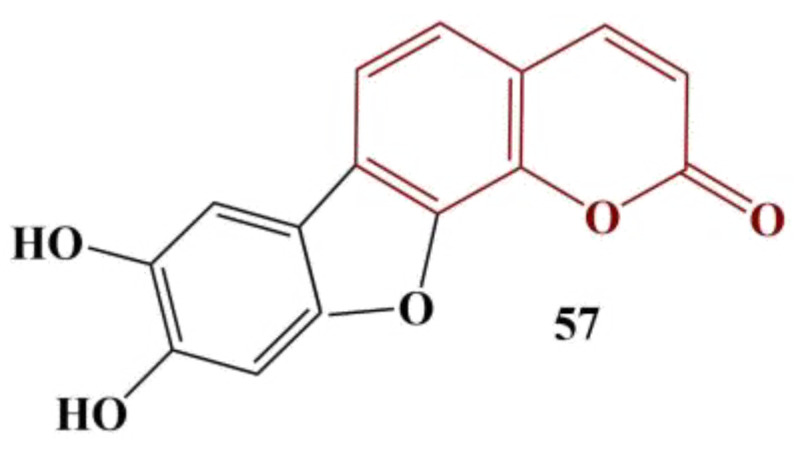
The natural coumarin, described in [57].

A novel, 7,8-disubstituted coumarin derivative (Figure 24) was isolated from the mushroom *Paxillus involutus* [57]. It manifested potent antioxidant activity against DPPH, with an IC_50_ of 16.3 μg/mL, compared to BHA (IC_50_ = 59.9 μg/mL).

**Figure 25:**
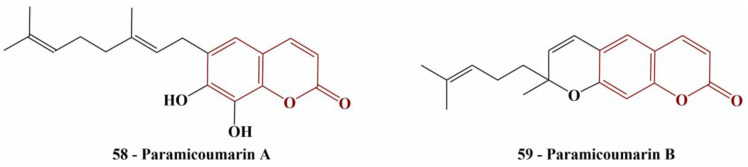
The natural coumarins, described in [58].

Two newly discovered coumarin compounds (named by the authors paramicoumarin A and B—Figure 25), isolated from Paramignya trimera roots were tested for scavenging activity against DPPH (0.075 mM, 1 h incubation in dark, room temperature) [58].

Paramicoumarin A did not exhibit significant DPPH-scavenging behavior and the authors decided not to calculate IC_50_. Paramicoumarin B had an IC_50_ of 101.9 μM—a better result than the standard, ascorbic acid (IC_50_ = 151.7 μM).

## 3. Antioxidant Properties of Coumarin Coordination Compounds

**Figure 26:**
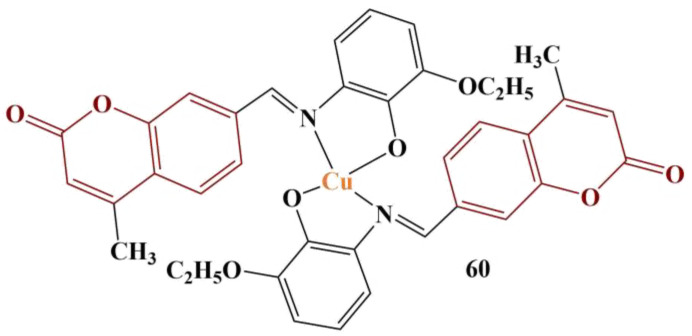
The Cu(II) complex, described in [59].

MacLean and colleagues designed and synthesized a number of Cu(II) complexes of coumarin-derived Schiff base ligands and tested them in order to establish potential pro- or antioxidant effects in the MCF-7 cell line [59]. ROS induction in the cell lines was estimated with the help of 2′,7′-dichlorodihydrofluorescin. Results were presented as “ROS induction (fold increase)” over a period of exposure (0–180 min). Most of the novel complexes did not manifest significant ability to induce ROS. Only one complex behaved as a prooxidant in a concentration-dependent manner (Figure 26).

This particular complex induces significant ROS generation at concentrations of at least 12.5 μM after cultivation of at least 2 h. Changing the ethoxy group in the phenyl ring for electron-donating methoxy/hydroxyl groups and adding electron-withdrawing nitro groups significantly diminishes the prooxidant effect.

**Figure 27:**
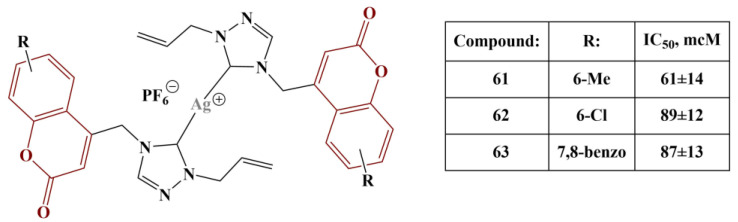
The Ag-NHC complexes, described in [60].

Geetha and colleagues incorporated coumarin with 1,2,4-triazole in order to generate silver *N*-heterocyclic carbene (NHC) complexes [60]. DPPH assay was employed to assess potential antioxidant activity. Bis-NHC-coordinated silver hexafluoprophosphate complexes showed better DPPH-scavenging activity compared to their acetate counterparts. Best results were observed when the coumarin skeleton was substituted as follows: 6-methyl, 6-chloro and 7,8-benzo (Figure 27) with moderate activities (IC_50_s between 61 and 87 μM). A negative effect was observed following 5,6-Benzo-substitution. Gallic acid was used for comparison (IC_50_ = 22 μM).

Bis-NHC-coordinated silver hexafluoprophosphate complexes showed roughly twice as good DPPH-scavenging activity compared to their acetate counterparts. Mono-NHC-coordinated silver bromide complexes exhibited intermediate activity (IC_50_s between 136 and 210 μM).

**Figure 28:**
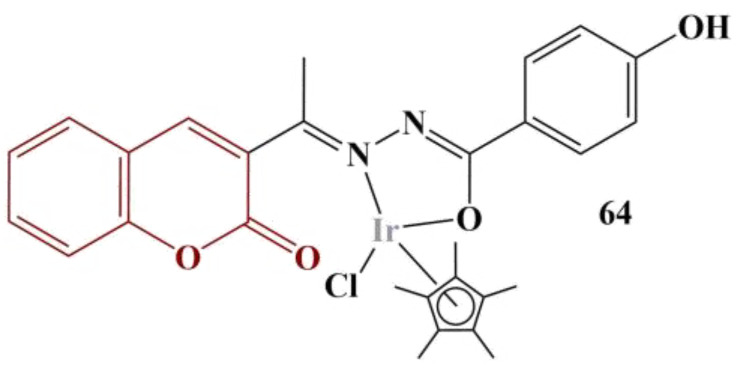
The most active complex, described in [61].

Nongpiur and colleagues [61] synthesized half-sandwich metal complexes with coumarin-N-acylhydrazone hybrid ligands. DPPH assay (0.004% in methanol, 30 min cultivation, dark conditions, room temperature) was applied in order to assess potential antioxidant activity. All tested compounds were dissolved in DMSO at a concentration of 1 mg/mL. Results were presented as DPPH Radical-Scavenging Activity at 1 mg/mL compared to a negative control (no substance present). Some of the novel compounds exhibited promising activities. The most active one, coordinating iridium (85.0% DPPH scavenging), is presented on Figure 28. Ascorbic acid was used as a standard (99.9% DPPH scavenging). Adding a diethylamino substitute at position 7 in the coumarin structure and substituting the 4-OH at the benzene ring for 3-OMe decreases antioxidant activity, though the complex is still a potent antioxidant (50.6% DPPH scavenging).

Changing the coordination center with ruthenium and substituting the arene ligand for *p*-cymene decreases scavenging activity down to 51.4%. Adding a 4,5-benzo-substituent to the coumarin structure decreases scavenging. Within the limits of the experiment, rhodium as a coordination center did not produce complexes with significant DPPH-scavenging activity.

**Figure 29:**
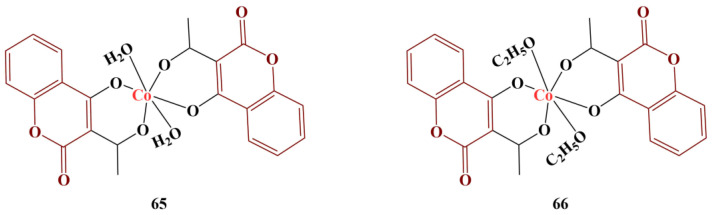
The Co(II) complexes, described in [62].

Bejaoui and colleagues synthesized 3-acetyl-4-hydroxycoumarin complexes (Figure 29) with cobalt(II) and tested them for antiradical activity [62] with the help of the DPPH assay.

Both complexes performed practically in an identical manner (IC_50_s of 0.022 μM and 0.021 μM). What the authors noted is that complexation with Co(II) improved the scavenging properties of the ligand: IC_50_ = 0.059 μM for the ligand itself. These compounds were much more potent than ascorbic acid (IC_50_ = 21 μM).

**Figure 30:**
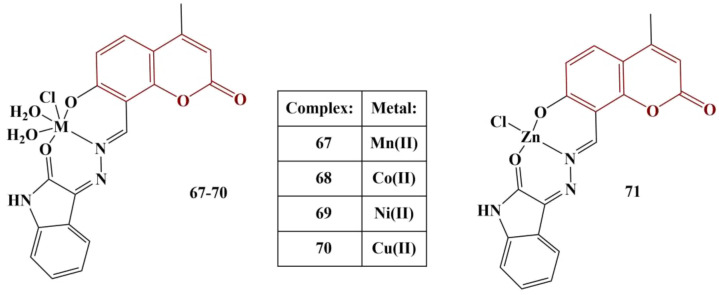
The coumarin–hydrazone complexes, described in [63].

Sunitha and colleagues synthesized a coumarin–hydrazone hybrid ligand [63] and coordinated it with several transition metal ions—Mn(II), Co(II), Ni(II), Cu(II) and Zn(II) (Figure 30). DPPH assay (0.004%, ethanol, 30 min cultivation, room temperature) was applied to assess potential antioxidant behavior at various concentrations, with vitamin C as a comparison substance. Results were presented as percentage inhibition compared to a negative control (no substance present). This experiment presents an opportunity to observe the impact of the coordination center on the radical-scavenging properties of the ligand.

The Co(II) complex scavenged 90.5% of DPPH, while the Ni(II) and Cu(II) complexes scavenged, respectively, 45.6% and 41.9% at 200μg/mL concentration. The ligand was practically inactive, while the rest of the complexes behaved as very weak scavengers (less than 13.3% at 200 μg/mL). The standard substance (ascorbic acid) had scavenged practically 100% at that concentration.

**Figure 31:**
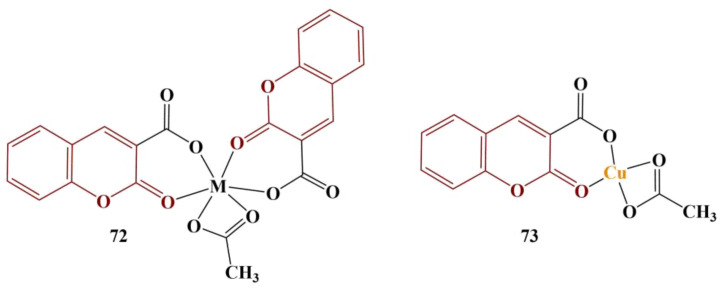
The coumarin-3-carboxylic acid complexes, described in [64].

Complexes of Ni(II), Cu(II) and Zn(II) with coumarin-3-carboxylic acid [64] were synthesized and tested with the DPPH assay (30μM, ethanol, 30 min incubation, room temperature, dark). Rutin was used as a reference compound. The three complexes are presented in Figure 31.

The ligand itself has a DPPH-scavenging effect close to zero. On the other hand, its complexes demonstrate enhanced scavenging effect that decreases in the following order: Cu(II) complex > Zn(II) complex > Ni(II) complex. Rutin was the best scavenger among all tested compounds.

**Figure 32:**
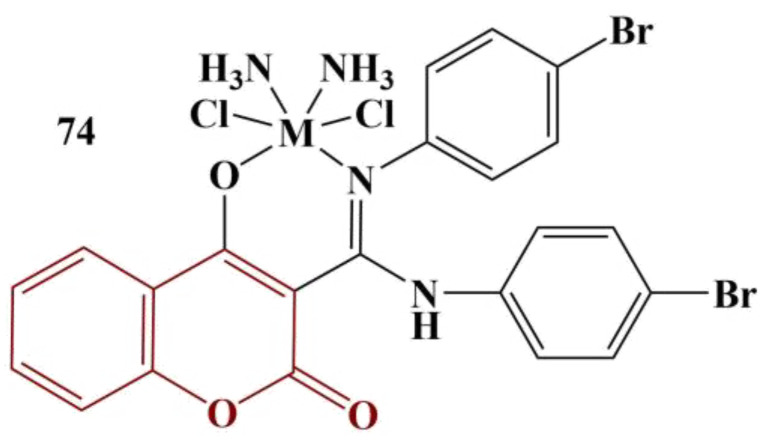
The complexes described in [65]; M = Cu(II)/Co(II)/Zn(II).

Belkhir-Talbi and colleagues synthesized Cu(II), Co(II) and Zn(II) complexes with *N,N*′-di(4-bromophenyl)-4-hydroxycoumarin-3-carboxamide (Figure 32) [65]. DPPH assay (4 mg/100 mL, ethanol, 1 h incubation, room temperature, dark) was applied to measure potential radical-scavenging activity.

The ligand itself behaves as a potent scavenger of DPPH (IC_50_ = 0.00049 mg/mL), more potent than the standard, ascorbic acid (IC_50_ = 0.00171 mg/mL). Coordination with the metal ions causes a massive decrease in antioxidant action. The Co(II) complex has the weakest activity (IC_50_ = 0.00701 mg/mL). The copper and zinc complexes have IC_50_ = 0.00386 mg/mL and 0.00383 mg/mL, respectively.

In another study, Cu(II), Co(II) and Zn(II) complexes of 3-(2-hydroxybenzoyl) coumarin were synthesized [66]. The metal:ligand molar ratio in all complexes was 1:1. DPPH assay (4 mg/100 mL, 1 h cultivation, room temperature, dark) was applied. The ligand exhibited very low scavenging activity (IC_50_ = 0.08664 mol/L). The scavenging effect of the zinc complex was nearly the same (IC_50_ = 0.08877 mol/L). The copper complex fared better, though still moderately (IC_50_ = 0.3841 mol/L), and the most active compound was the cobalt complex (IC_50_ = 0.02666 mol/L). The control substance, ascorbic acid, had a significantly stronger scavenging effect (IC_50_ = 0.00077 mol/L). The authors proposed that the low oxidation potential of these ions allows them to lose an electron in order to reduce DPPH to its non-radical form.

A series of coumarin–pyrazole complexes with Ru(II) were applied to laboratory mice with diabetes mellitus [67]. Lipid peroxidation in the serum, liver and kidney was suppressed compared to the untreated diabetes group. Additional investigations revealed that treated animals had elevated GSH levels compared to untreated animals. SOD, CAT and GPx levels (presented as Units/mL for SOD and CAT and Units/L for GPx) were elevated compared to untreated diabetic animals. The authors conclude that the novel complexes exhibit protective, antioxidant effects.

**Figure 33:**
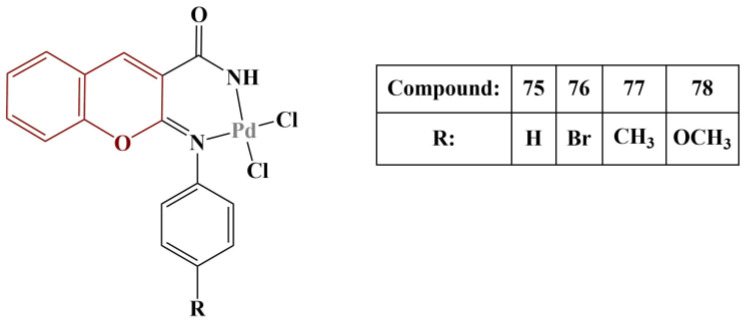
The iminocoumarin complexes, described in [68].

Novel Pd(II) complexes with iminocoumarin ligands were synthesized and tested for antioxidant activity [68] (Figure 33).

DPPH assay (30 min incubation, room temperature) was applied. All compounds exhibited a modest scavenging effect (IC_50_ > 1000 μg/mL). The most active scavenger was Compound 78 (IC_50_ = 471,55 μg/mL), followed by Compound 77 (IC_50_ = 681,83 μg/mL). Ascorbic acid was used as a standard (IC_50_ = 380,96 μg/mL). The authors surmised that the electron-rich coordination center reinforces electron density in the ligand molecules, thus improving their scavenging ability.

**Figure 34:**
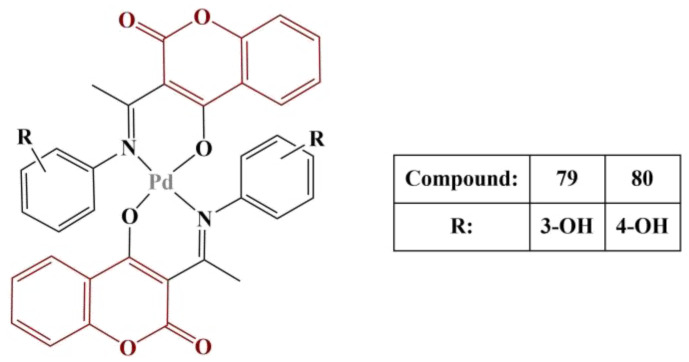
The Pd(II)-hydroxycoumarin complexes, described in [69].

Two more novel Pd(II) complexes with 4-hydroxycoumarin derivatives (Figure 34) were tested for antioxidant activity [69].

Results from the ABTS assay (2 mM ABTS, 15 μM hydrogen peroxide, 15 μM horseradish peroxidase) showed that Compound 79 manifested observable scavenging activity (59 μM AAE—ascorbic acid equivalent). The corresponding ligand had a lower activity of 19 μM AAE. All other compounds had activity below the detection limit of the method. Both complexes suppressed Fenton-generated hydroxyl radicals at 5 μM, the effect of Complex 79 being stronger than Complex 80 (68% suppression versus 62% suppression). The corresponding ligands had no observable effect on that model system.

A novel tridentate ligand 4-(2-hydroxy benzylidene acetohyrazide)-7-hydroxy coumarin was coordinated with Sm(III) and Eu(III) by Elsenety and colleagues [70]. The metal:ligand molar ratio in the complexes was 1:2. The authors performed molecular docking on the novel compounds, including the respective ligand, which revealed a possibility that they may bind to the active site of the superoxide-producing enzyme XO. Phosphonic acid mono-(2-amino-5,6-dimercapto-4-oxo-3,7,8a,9,10,10a-hexahydro-4*H*-8-oxa-1,3,9,10-tetraaza-anthracen-7-ylmethyl)ester (MTE) and flavin adenine dinucleotide (FAD) were used as comparison in the docking study. Calculations revealed that the novel ligand may interact with chain C of XO compared to the reference MTE.

Benarous and colleagues [71] investigated series of complexes of bis-coumarins with Ln(III), Ce(III) and Nd(III) for inhibitory activity on HXO and BXO. All lanthanide complexes investigated exhibited significant inhibitory activity (IC_50_ values between 12.91 nm and 23.83 nm), resembling that of the positive control allopurinol (IC_50_ = 19.98 nm).

**Figure 35:**
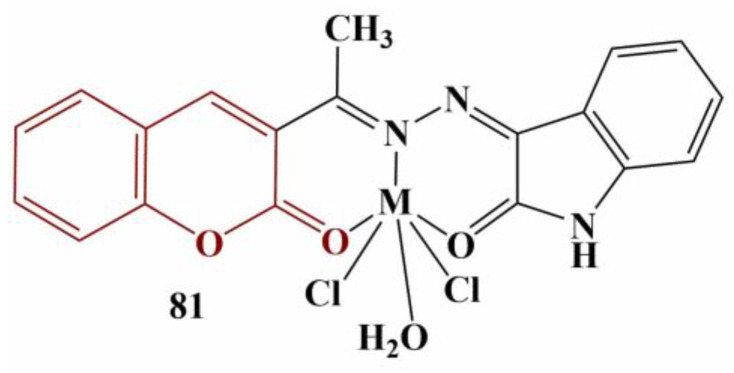
The coumarin–isatin hybrids, described in [72]. M = Mn(II)/Co(II)/Ni(II)/Cu(II).

Complexes of 3-acetylcoumarin-isatin monohydrazone with Mn(II), Co(II), Ni(II), Cu(II) and Zn(II) with the common structure displayed in Figure 35 were synthesized and tested for possible antioxidant activity with DPPH assay [72]. Results were presented as percentage of inhibition compared to a negative control (no substance present).

The strongest scavenger at 100 μg/mL was the cobalt complex (93.14% scavenging), followed closely by manganese (90.76%), zinc (89.34%), the free ligand (72.85%), nickel (49.00%) and copper (48.04%). The standard substance (ascorbic acid) scavenged 73.18% at that concentration.

A Schiff base ligand was synthesized from 3-acetylcoumarin and 2-methylbenzylhydrazinecarbodithiolate and coordinated with Co(II), NI(II), Cu(II) and Zn(II) [73]. The metal:ligand ratio was 1:1. DPPH assay (0.004%, methanol, 30 min cultivation, dark, 28 centigrade) revealed that coordination with the metal ions improves the antioxidant effect of the ligand. Results were presented as percentage inhibition compared to a negative control (no substance present). The improvement of scavenging by the metal ions increased in the following order: Ni < Co < Zn < Cu. At a concentration of 1 mg/mL, the Cu(II) complex inhibited 45% of DPPH and the standard substance, ascorbic acid, at the same concentration inhibited 65%.

**Figure 36:**
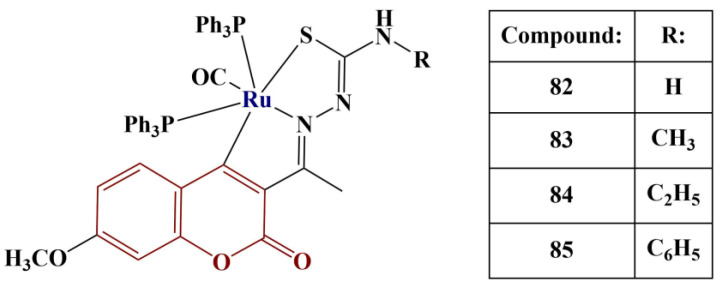
The Ru(II)–Schiff base ligand, described in [74].

A series of 3-acetyl-7-methoxy coumarin Schiff base ligands and their Ru(II) metalates (Figure 36) were tested for radical-scavenging activity [74].

All respective Schiff base ligands manifested moderate DPPH-scavenging activity—IC_50_ values were between 67.28 μM and 91.21 μM. Once coordinated with ruthenium(II), their respective complexes exhibited about tenfold lower IC_50_ values (IC_50_ values between 5.28 μM and 7.39 μM). Compound 84 and its respective ligand performed best compared to their counterparts. In this model system, the IC_50_ of the standard substance ascorbic acid was 98.72 μM, emphasizing the effectiveness of the complexes as radical scavengers.

**Figure 37:**
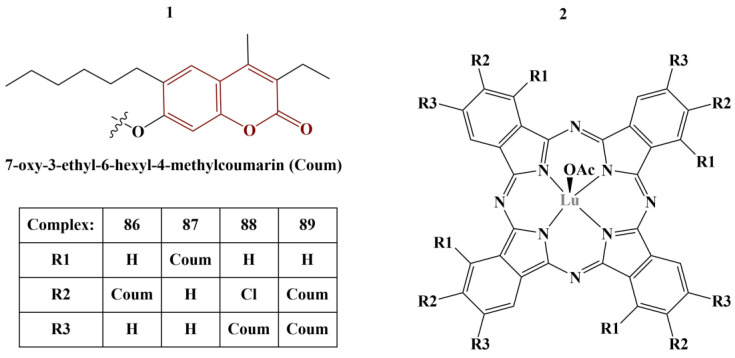
The structure of the coumarin moiety (**1**) and the complexes (**2**), described in [75].

A series of phthalocyanine–coumarin hybrids were synthesized, coordinated with lutetium(III) (Figure 37) and tested for ABTS-scavenging, FRAP and CUPRAC [75].

ABTS-scavenging activity was highest with Complexes 88 and 89, IC_50_ being 120.34 and 188.73 mM Trolox/mg, respectively (higher value is better), much higher than the standard (BHA IC_50_ = 52.63 mM Trolox/mg). On the contrary, the activity of the other two compounds was measured to be 20 times weaker. FRAP assay showed Compounds 87 and 89 to be the most potent (IC_50_ = 0.375 and 0.356 mM Fe^2+^/mg, respectively), about 2–3 times more potent than Compounds 86 and 88, but weaker than the standard BHT ((IC_50_ = 1.1 mM Fe^2+^/mg). Compounds 87 and 89 were also the stronger copper-reducing agents (IC_50_ = 2.040 and 1.775 mM Trolox, respectively), about 10 times as potent as 86 and 88, but less potent than vitamin C (IC_50_ = 2.70 mM Trolox).

## 4. Discussion and Conclusions

The coumarin structure offers a wide range of opportunities for developing novel medicinal substances. Antioxidant action is an important component of the safety profile of potential drug molecules. The results presented herein are derived from a variety of antioxidant assays, performed under somewhat non-uniform sets of conditions on a heterogenous mix of chemical structures. To “add confusion to the chaos”, experimental data from different research teams are frequently expressed in disparate units of measurement, making direct comparisons not entirely suitable. Despite that, a certain set of “rule of thumb” conclusions can be made:

- “Classical rules” still apply—adding electron-donating substituents (particularly hydroxy and amino groups at positions 4 and 7) to the coumarin structure tends to improve the antioxidant effect, as well as chlorine at position 6. Adding an ortho-dihydroxy catecholic motif to coumarin ring B increases antioxidant activity. The most prominent impact seems to stem from 7,8-dihydroxy substitution. On the other hand, 5,7-dihydroxy substitution seems to improve metal chelation.
- Adding resonance-contributing functionalities (conjugated double bonds, aromatics, etc.) tends to improve the antioxidant effect.
- Nitrogen-bearing functionalities at position 3 (Schiff base, hydrazone, hydrazide, etc.) tend to contribute to the radical-scavenging effect and also present opportunities for polydentate chelation of metal ions.
- Adding electron-donating groups (halogen, methyl, hydroxyl) to aromatic structures incorporated in the coumarin substituents more often than not improves radical scavenging.
- Chelation with transition metals can improve the radical-scavenging effect of ligands. From the research presented herein, a tentative “rule of thumb” would point to Co(II), Cu(II), Ni(II) and Pd(II) as coordination centers that tend to generally have a positive effect.

In the course of completing the present review, several trends of development when it comes to coumarin substances with antioxidant action have been noted by the authors as possible future prospects. Many of the novel substances discussed were investigated for antioxidant properties as a secondary biological activity (the primary being antifungal, antimicrobial, anti-inflammatory, antidiabetic, etc.). Promising results have been obtained by adding coumarin moieties to polysaccharides (chitosan and inulin), yielding polymer compounds with improved antioxidant activity. Such novel compounds show the potential to play a future role in a number of fields of science and industry—medicine, pharmaceuticals and food to name a few. Results in that direction are still but a few; however, the potential benefits cannot be overestimated. Another prospective field of future research is the synthesis of coumarin ligands with biological activity (antifungal, antibacterial, antidiabetic, anticancer) and their complexation with transition metals. Such coordination seems more often than not to cause an improvement in biological activity. Considering the great variety of physiological activities associated with the coumarin structure, it is safe to propose that further studies be carried out. Adding coumarin moieties to photosensitizers seems to be another interesting area of future research, yielding novel agents for photodynamic therapy by combining intrinsic antioxidant action with improved singlet oxygen quantum yield in targeted tissues.

The authors have noted the great variety of antioxidant assays that have been applied by researchers, the most prominent involving DPPH, ABTS, FRAP, CUPRAC and Fenton reaction. In terms of antioxidant assays, we can definitely say that “the more, the better”.

Each type of assay adds a different angle to the observed overall picture of potential antioxidant behavior. Keeping in mind that such experiments can be costly, both in terms of time and effort, it is understandable that in many cases relatively few types of tests have been applied by researchers. It is the authors’ observation that greater investigation into the scavenging of the superoxide and hypochlorite reactive species may be particularly useful due to their physiological relevance, especially in terms of processes such as inflammation and immune response.

In conclusion, the authors would like to offer a brief summary of the data on some of the more promising compounds included in the review, as shown in Table 2. It offers the number of the compound, the general chemical structure, types of assays performed and the standard substance for comparison and results from the assays, together with those for the standard compound.

## Author Contributions

Conceptualization, I.K. and L.S.; investigation, L.T.; resources, L.T.; supervision, I.K. and L.S.; visualization, L.T.; writing-original draft, L.T.; writing—Review & editing, I.K. All authors have read and agreed to the published version of the manuscript.

## Figures and Tables

**Table 1:** Some examples of RS generation in living organisms.

Source of RS	Type of RS	Site of Production	Function
Nicotinamide adenine dinucleotide (NADH) dehydrogenase (Complex I)	superoxide	Mitochondria	Electron transport during oxidative phosphorylation.
Coenzyme Q (Complex III)	superoxide	Mitochondria	Electron transport during oxidative phosphorylation.
Nicotinamide adenine dinucleotide phosphate (NADPH) oxidase (NOX)	superoxide	Cytosol, membranes of various organelles	Immune response, phagocytosis.
Xanthine oxidase	Superoxide, hydrogen peroxide	Liver, intestines	Catalytic conversion of hypoxanthine to xanthine and uric acid.
Nitric oxide synthase	Nitric oxide (NO), superoxide	Cytosol, cellular membranes	Synthesis of NO from L-arginine.
Myeloperoxidase	Hypochlorous acid	Neutrophils	Synthesis of hypochlorous acid during respiratory burst.
5-lipoxygenase	Indirect action	Immune cells	Leukotriene production, causing NOX stimulation and RS generation.
Peroxynitrite production	Peroxynitrite ion	Phagocytes	Produced from superoxide and NO, phagocytosis.
Fenton reaction and Haber–Weiss chain	Hydroxyl/hydroperoxyl radicals	Wherever transition metal ions such as iron and copper come in contact with hydrogen peroxide and/or superoxide.	Mostly associated with pathologies.

**Table 2:** Brief summary of the data on some well-performing compounds, described in the present review.

Compound Number	Chemical Structure	Assay Type/Standard	Result (Compound/Standard)
3	4-methylcoumarin	DPPH/Trolox	EC_50_ = 150.99 μM/243.39.99 μM
		ABTS/Trolox	EC_50_ = 39.98 μM/83.50 μM
		Galvinoxyl/Trolox	EC_50_ = 13.19 μM/20.86 μM
4	4,7-dimethylcoumarin	DPPH/ascorbic acid (AA)	IC_50_ = 10 μg/mL/33.48 μg/mL
7	3-phenylcoumarin	DPPH/Trolox	EC_50_ = 64.27 μM/93.19 μM
		FRAP/Trolox	EC_50_ = 2.28 μM/1.0 μM
		CUPRAC/Trolox	EC_50_ = 2.44 μM/1.0 μM
13	Coumarin–Schiff base	Fenton reaction/AA	IC_50_ = 2.61 μM/300 μM
18	Coumarin–thiosemicarbazone	DPPH/AA ABTS/Trolox	IC_50_ = 7.1 μM/18.6 μM IC_50_ = 9.0 μM/13.0 μM
23	Coumarin-3-sulfonamide	DPPH/AA	IC_50_ = 4.30 μg/mL/2.83 μg/mL
29	Coumarin-1,3,4-oxadiazole	DPPH/AA Fenton reaction/butylated hydroxytoluene (BHT)	IC_50_ = 19.47 μM/23.80 μM IC_50_ = 19.47 μM/36.05 μM
31	Coumarin–hydroxytyrosol	DPPH/BHT ABTS/BHT	IC_50_ = 26.58 μM/521.99 μM IC_50_ = 30.31 μM/127.07 μM
41	Coumarin–thiazine	DPPH/AA	IC_50_ = 35.35 μg/mL/36.22 μg/mL
53	Natural 7-8-dihydroxycoumarin	DPPH/BHT Fenton reaction/AA	IC_50_ = 48.20 μM/46.29 μM IC_50_ = 52.84 μM/50.33 μM
58	Natural 7-8-disubstituted coumarin	DPPH/butylated hydroxyanisole (BHA)	IC_50_ = 16.3 μg/mL/59.9 μg/mL
59	Natural 6,7-disubstituted coumarin	DPPH/AA	IC_50_ = 101.9 μM/151.7 μM
65,66	Co(II) complexes with 3-acetyl-4-hydroxycoumarin	DPPH/AA	IC_50_ = 0.022 μM/21 μM
74 (the free ligand)	4-hydroxycoumarin-3-carboxamide	DPPH/AA	IC_50_ = 0.00049 mg/mL/0.00171 mg/mL
81	Co(II) complex with coumarin–isatin hybrid	DPPH/AA	Scavenging at 100 μg/mL= 93.14%/73.18%
84	Ru(II) complex with coumarin–Schiff base ligand	DPPH/AA	IC_50_ = 5.28 μM/98.72 μM

## Data Availability

Not applicable.
